# Antimicrobial Photoinactivation Approach Based on Natural Agents for Control of Bacteria Biofilms in Spacecraft

**DOI:** 10.3390/ijms21186932

**Published:** 2020-09-21

**Authors:** Irina Buchovec, Alisa Gricajeva, Lilija Kalėdienė, Pranciškus Vitta

**Affiliations:** 1 Institute of Photonics and Nanotechnology, Faculty of Physics, Vilnius University, Sauletekio av. 3, LT-10257 Vilnius, Lithuania; pranciskus.vitta@ff.vu.lt; 2 Department of Microbiology and Biotechnology, Institute of Biosciences, Life Sciences Center, Vilnius University, Sauletekio av. 7, LT-10257 Vilnius, Lithuania; lilija.kalediene@gmc.vu.lt

**Keywords:** antibacterial photoinactivation, natural photosensitizers, spacecraft biofilm decontamination

## Abstract

A spacecraft is a confined system that is inhabited by a changing microbial consortium, mostly originating from life-supporting devices, equipment collected in pre-flight conditions, and crewmembers. Continuous monitoring of the spacecraft’s bioburden employing culture-based and molecular methods has shown the prevalence of various taxa, with human skin-associated microorganisms making a substantial contribution to the spacecraft microbiome. Microorganisms in spacecraft can prosper not only in planktonic growth mode but can also form more resilient biofilms that pose a higher risk to crewmembers’ health and the material integrity of the spacecraft’s equipment. Moreover, bacterial biofilms in space conditions are characterized by faster formation and acquisition of resistance to chemical and physical effects than under the same conditions on Earth, making most decontamination methods unsafe. There is currently no reported method available to combat biofilm formation in space effectively and safely. However, antibacterial photodynamic inactivation based on natural photosensitizers, which is reviewed in this work, seems to be a promising method.

## 1. Introduction

A spacecraft is a closed system inhabited by microorganisms originating from life support systems, cargo, and most importantly, crewmembers [1,2,3]. Microorganisms found in the spacecraft are exposed to unique selective pressures such as microgravity, space radiation, elevated carbon dioxide levels, hypomagnetic conditions, and continuous human habitation [4]. It has been shown that sometimes these conditions provoke unusual bacterial traits, such as the faster acquirement of antibiotic, chemical, and physical resistance; faster biofilm formation; and altered growth kinetics compared to Earth conditions [5,6,7]. The microbiome of the confined spacecraft environment needs to be examined constantly to identify the species of microorganisms that can accumulate in this unique environment, which can change and survive under stress conditions as free-living cells or most importantly as biofilms, and which can influence human health, spacecraft infrastructure, and material integrity. Such findings could help to find the best way of controlling microorganisms in such environments and enable safe, long-duration space missions. In the period since the first taxonomical and quantitative studies of spacecraft microbiomes, culture-dependent and later culture-independent studies have revealed a broad range of microbial loads. The most comprehensive study of the microbes that are able to survive in spacecraft was carried out at the International Space Station (ISS). All of the bacteria detected in the spacecraft can be divided into three groups: (1) potentially pathogenic bacteria; (2) technophilic bacteria, and (3) bacteria that do not affect crewmembers’ health or the equipment. Analysis of the scientific literature describing the microbiological bioburden of spacecraft has shown that in the majority of the studies, certain bacterial taxonomic groups prevail among others. The most abundant airborne bacteria in spacecraft, as determined by conventional culture methods and cultivation-independent molecular methods, are identified as *Enterobacteriaceae*, *Staphylococcus* spp., *Bacillus* spp., *Corynebacterium* spp., and *Propionibacterium* spp. The most frequent potable water-system species in most cases were reported to belong to *Methylobacterium* spp., *Sphingomonas* spp. (particularly *Sphingomonas paucimobilis*), *Cupriavidus* spp., *Chryseobacterium* spp., and *Ralstonia* spp. [8,9,10,11,12]. Most of the above mentioned bacteria are human-associated, usually found on human skin, and are determined to be responsible for the formation and diversity of spacecraft microbiota. The most significant equipment damage was reported to be caused by *Bacillus* spp. and several mold species [3,13].

The most abundant bacteria found in the spacecraft are most likely to form biofilms. Bacterial biofilms in the spacecraft usually form on hard-to-clean, moist areas such as piping and equipment behind panels, electrical connectors, thermal control system radiators, the water recycling system, and the rubber of hatch locks, etc., which could be dangerous for both spacecraft equipment and human health [3,4,11,13]. However, some recent studies suggest that ISS conditions are selective but do not alter microbial characteristics relevant to human health. Therefore, according to the latest research on the development of bacterial biofilm in space conditions, this bacterial mode of growth is currently considered to pose a higher threat to the spacecraft material integrity than the crewmembers’ health [1,3,14]. Nevertheless, the fact that biofilms were shown to form more quickly in spaceflight conditions and to form thicker, different unique structures than on Earth [3,5,6], the impact of the bacterial biofilms on the spacecraft’s crew health must be studied in more detail, using suitable techniques in order to draw more definitive conclusions.

Since the environment of space in some cases alters bacterial biofilms through increasing their chemical and physical resilience, the development of alternative technologies could be the first efficient step towards the safe reduction of this critical problem in spacecraft. In this context, the antimicrobial photoinactivation (API) approach seems to be one of the most promising strategies. API is a biophotonic technology involving the employment of a photoactive compound (a photosensitizer (PS)) that selectively accumulates in the target cells, which are then illuminated [15,16]. The interaction of the PS and light in the presence of oxygen results in a plethora of cytotoxic reactions. Usually, after light excitation, the triplet-state of the PS interacts with molecular oxygen, electron donors, or acceptors and can produce reactive oxygen species (ROS), thereby triggering photo-oxidative reactions that initiate various forms of cellular damage, thus destroying the bacterial cells [17].

The antibacterial efficiency of API is influenced by many factors, but especially by the physicochemical properties of the PS chosen. Many significant advances have been made in PS research during the last 20 years. It has been reported that API is effective in eradicating both planktonic cells and biofilms of bacteria. A wide range of compounds of different structures can be applied, and the efficacy of some PSs has already been evaluated for the eradication of biofilms. Generally, phenothiazinium dyes (e.g., Methylene Blue, Toluidine Blue), porphyrins (e.g., TMPyP), and xanthene dyes (e.g., Erythrosine and Rose Bengal) have been used for API applications [18,19]. There are several advantages of the API method for the control of bacteria biofilms in specific, confined spacecraft environments: (1) API belongs to a multitarget process, so no development of bacterial resistance occurs; (2) microbial killing is rapid (occurs within seconds); and (3) broad-spectrum action removes the need to identify the particular pathogen (identification is difficult to perform in the space environment). Unfortunately, not all mentioned dye-based PSs are effective and can be unsafe to use in spacecraft. For the application of API in the spacecraft environment, it is necessary to use PSs which are chemically pure and easy to produce, water-soluble, and non-bleaching, with a stable shelf-life, and most importantly which are safe, meaning that they must not alter the crewmembers’ health [19,20]. Compared with synthetic PSs, naturally occurring agents are of great interest due to the variety of their molecular structure and specific biological activities. Analysis of the literature regarding the application of API to control bacterial biofilms shows that a large number of natural products and their derivatives have significant photodynamic activities, and some of them have been clinically applied. There are several main natural products that can be used in API—some of the most commonly used are curcumin (CUR), riboflavin (RF), perylenequinones (hypericin (Hyp), hypocrellin), psoralens, and chlorophyll derivatives (sodium chlorophyllin (Chl)) [16,18,21]. The emergence of new detection and analysis technologies raises the possibility of the rapid identification of potent PSs derived from natural products, and the extension of the application of already known natural agents.

## 2. Biofilms in Spacecraft

Bacterial free-living (planktonic) cells are able to switch to the biofilm mode of growth—a sessile and structured three-dimensional community of microorganisms on a surface, encapsulated in a self-formed matrix made of extracellular polymeric substances (EPS) [22,23]. Biofilm formation is considered to be a default mode of growth, enabling bacteria to sequester in a nutrient-rich area, utilize the cooperative benefits of living in a community, and protect themselves from harmful conditions [24]. Usually, these bacterial consortia are associated with moist, hard-to-clean surfaces. The properties of microorganisms living within a biofilm generally differ substantially from those of microorganisms of the same species existing independently [25]. The presence of biofilm makes bacteria more resilient and non-responsive to the treatments currently used [26]; once established, biofilms become highly resistant to various chemicals (antibiotics, antimicrobial disinfectants, etc.) and physical effects. Therefore, biofilm-associated microbial harm poses a severe threat not only in the hospital and industrial settings on Earth but also in confined areas such as the ISS or other spacecraft. Although spacecraft are assembled in cleanrooms in order to minimize microbial contamination, some microorganisms withstand and survive decontamination methods applied in ultraclean facilities. These microorganisms are more often referred to as extremophiles that can deteriorate abiotic surfaces and do not endanger the crew’s health. A greater risk to crewmembers in the spacecraft may arise from microbes originating from their own microbiota, water, and food supplies [11,14]. The diversity and abundance of microorganisms from surfaces, air filters, and potable water systems on the ISS have been studied in detail, and human-associated bacteria were found to be the most frequently isolated, therefore comprising a set of microorganisms which are most likely to form biofilms in spacecraft [8,27,28,29,30,31,32,33,34,35]. Isolates from the ISS are able to grow as biofilms under standard laboratory conditions, suggesting that the capacity to form complex communities on surfaces and interfaces provides a competitive advantage aboard spacecraft [11]. Biofilms may play an important role in human infections and create a potential threat towards material integrity, especially in terms of long-term space missions, since the extent and complexity of microbial contamination are increasing with time [1,11]. Thus, the microbiome of the closed space environment needs to be examined comprehensively in order to identify microorganisms that can accumulate in this unique environment, survive under stress conditions in biofilms, and influence both human health and key equipment of the spacecraft. Such findings could help to find the best way of controlling biofilms in such environments and enable safe space missions that will lengthen over time due to the progress and expansion of human activity in space [3].

### 2.1. Effects of Microbial Biofilms on Spacecraft Crew Health

Microbial biofilm growth, increasing the risk of human illness and sometimes damaging key equipment such as spacesuits, water recycling units, radiators, and navigation windows, has been observed in Soviet/Russian (Salyut and Mir), American (Skylab) space stations, and the ISS. It is important to emphasize that spacecraft such as the ISS are confined and closed habitats, exposed to unique conditions such as cosmic radiation, microgravity, and hypomagnetism, and these conditions have a substantial effect on human and spacecraft microbiota [3,4,36,37]. Space conditions can affect the immune responses of the crewmembers by altering respiratory, gastrointestinal, and nasal bacterial microbiota, which may represent health risks for the crew. Thus, bacterial biofilms that form on the indoor equipment surfaces of spacecraft may pose an even a more significant threat to the human health [4]. In some of the early space station missions like Mir and Skylab, spaceflight crew members became ill in space. In-flight cross-contamination with *Staphylococcus aureus* and other pathogens in the upper respiratory tract of astronauts has since been reported [38]. In the US segment of the ISS, from December 2011 to July 2012, allergic responses to the cabin environment and dust in the Node 3 cabin were also reported. The latter findings led to a comprehensive study of particulate and debris samples from the ISS [2,11]. However, more recent studies suggest that ISS conditions are selective, but do not alter microbial characteristics relevant to human health [1]. Mora et al. (2019) reported the results of the ISS experiment ”Extremophiles”, which involved the study of microbial communities from several areas aboard the ISS at three time-points. Scientists evaluated the diversity, distribution, functional capacity, and resistance profile, using a combination of cultivation-dependent and cultivation-independent methods. Results showed that the ISS microbial communities are highly similar to those present in ground-based confined indoor environments and are subject to fluctuations. However, a core microbiome persists over time and across locations. The genomic and physiological features selected by the ISS conditions do not appear to be directly relevant to human health, although adaptations towards biofilm formation and surface interactions were observed. Therefore, results did not show a direct cause for concern about crew health, but indicated a potential threat towards spacecraft infrastructure material integrity in moist areas [1]. Some previous studies showed that *S. aureus* in low-shear modeled microgravity (LSMMG) conditions may not respond to environmental stresses as well as under normal gravity conditions, and the accumulated data on the impact of microgravity indicate that staphylococci display a biofilm phenotype with reduced virulence characteristics [11,39]. Further evidence that Gram-positive bacteria, Gram-negative bacteria, and yeasts are less virulent than controls when grown under microgravity conditions has emerged from comprehensive studies of the capacity of *Listeria monocytogenes*, methicillin-resistant *S. aureus* (MRSA), *Enterococcus faecalis*, and *Candida albicans* to kill *Caenorhabditis elegans* nematodes at the larval and adult stages on the ISS and under clinorotation [40]. In a study by O’Rourke et al. (2020) genomes of the opportunistic pathogens *Burkholderia cepacia* and *Burkholderia contaminans* (genomovars of the *B. cepacia* complex), which are frequently cultured from the potable water dispenser (PWD) of the ISS were sequenced and phenotypic assays to characterize these *Burkholderia* isolates were conducted. Results showed that all ISS-derived isolates exhibited antibiotic sensitivity similar to that of the terrestrial reference strains, and minimal differences between isolates were observed. With a few exceptions, biofilm formation rates were generally consistent across each genomovar. Overall, O’Rourke et al. (2020) concluded that while the populations of *Burkholderia* present in the ISS PWS each maintained virulence, they probably were not more virulent than those that might be encountered on Earth and were found to be susceptible to clinically used antibiotics [41].

Nevertheless, some studies (Section 2.3) have shown the contrary, that the virulence of some bacteria, as well as antibiotic resistance, can be enhanced in space conditions. Therefore, for now, some inconsistency regarding biofilm-forming microbial isolates and their possible impact on human health in space conditions exists. The potential impact of the development of diverse microbial populations is unclear. Although some studies indicate that biofilms pose a more significant threat as biodestructors of abiotic surfaces of different spacecraft equipment rather than a health-threat for the space crew, this potential problem should not be ruled out and should be studied in more detail.

### 2.2. Microbial Biofilm Damage and Corrosion of Indoor Spacecraft Equipment

Microbial contamination of the indoor surfaces of spacecraft may damage materials and decrease the efficiency of equipment [3,14]. Onboard the Salyut 6 spacecraft, biofilms were found on the piping and equipment behind panels. On Salyut 7, electrical connectors, the thermal control system’s radiator, the water recycling system, and the rubber of hatch locks were found to be contaminated by microbial biofilms. On the Mir station, biofilms affected the navigation window, air conditioning, oxygen electrolysis block, water recycling unit, suit headphones, and the thermal control system [3,4,11,13]. The main effect of the destruction of a navigation window onboard Mir was attributed to *Bacillus polymira*, *Penicillium rubens*, and *Aspergillus* sp. [3,13]. A study of microorganisms in the Russian Mir space station revealed 234 different species of bacteria and fungi. Most of the isolated fungi were potential biodestructors of polymers and thus presented a potential hazard to structural materials and components of several spacecraft systems [3]. Most of the organisms in the ISS water supplies were reported to belong to Gram-negative *Proteobacteria*, such as *Methylobacterium*, *Sphingomonas*, *Ralstonia*, and *Pseudomonas* [42,43,44]. Novikova et al. (2006) performed a six-year study aiming to characterize the microbiome present onboard the ISS. They found that bacterial concentrations of up to 1 × 10^2^ CFU × mL^−1^ were found in potable water, with *Sphingomonas* sp. and *Methylobacterium* sp. being the dominant genera. Samples collected from surfaces showed *Staphylococcus* sp. to be the predominant bacteria. Airborne bacteria were also quantified, with *Staphylococcus* sp. again being the dominant genus.

Organic acids synthesized by microorganisms can degrade metallic surfaces, which can lead to hardware malfunctioning and short circuits [8]. Further studies emphasized that spacecraft planktonic and biofilm microbial diversity is strongly influenced by human skin-associated microbes [1,2,3]. The latter has also been confirmed by a metagenome profile study [33]. On the ISS, biofilms were also detected on rubber seals, viewing windows, and on different hardware surfaces [14], with a variety of pathogens isolated from the ISS forming robust biofilms. Most biofilm formers were staphylococci and enterococci [4,14].

### 2.3. Biofilm Formation Investigation during Spaceflight under Assessed Conditions

Interestingly, some investigations have revealed that bacterial growth, biofilm formation, and even virulence can be enhanced under spaceflight conditions [3,45,46,47]. Several studies have shown that biofilms actually grow and accumulate more in space than on Earth. They form faster and therefore become resistant to antibiotics more quickly, and they make thicker structures and have different forms [5,6,7]. To date, only a few investigations have been conducted in space to evaluate biofilm formation. One of the first experiments was done using *B. cepacia* [48] as a model organism, which was previously isolated from the water system of Space Shuttle STS-81 (using the European Space Agency’s PHORBOL cassette hardware onboard the Space Shuttle STS-81 mission). Samples of isolated *B. cepacia* were grown in conditions simulating untreated water, wastewater, and disinfected potable water. To simulate these conditions, sterile reagent grade water, tryptic soy broth (TSB), and iodine solution were used, respectively. The results showed that the spaceflight water-grown bacteria had a biofilm plate count (CFU × mL^−1^) five times higher than controls on Earth. On the other hand, the spaceflight TSB-grown culture biofilm population was one-quarter of the ground controls. Analyses of the water- and iodine-grown planktonic bacteria showed a 3.5- and 2-fold increase in CFU × mL^−1^ with respect to matched ground controls. Pyle et al. (1999) concluded that spaceflight enhanced bacterial growth and diminished disinfectant sensitivity in some conditions [48].

McLean and colleagues (2001) studied *Pseudomonas aeruginosa* biofilm formation under microgravity conditions [49]. Type III Osmotic Dewatering hardware was used for the evaluation. Cultures of *P. aeruginosa* were exposed to 0.2-μm polycarbonate membranes, allowing them to form biofilms for either one or eight days. Post-flight confocal laser scanning microscopy revealed that the biofilms formed during the spaceflight had no morphological differences compared to ground samples. However, spaceflight had experimentation limitations: the experiment consisted of four spaceflight cultures, but only two were used for microscopic analysis [49].

Another spaceflight biofilm formation investigation was conducted by Kim et al. (2013). The investigation consisted of two experiments, which were done using BioServe Space Technologies’ Fluid Processing Apparatus (FPA) during the STS-132 and -135 missions. The experiments assessed the impact of phosphate, carbon source, bacterial motility, and oxygen availability on biofilm formation of three *P. aeruginosa* strains in space. It was concluded that the number of viable cells, biomass and mean biofilm thickness was increased in space, regardless of phosphate concentration or carbon source. Moreover, interestingly, *P. aeruginosa* biofilms formed in space exhibited a “column-and-canopy” structure, as opposed to the flat structures observed on the ground controls [5,6].

Furthermore, it was determined that *Escherichia coli* cells grown under low shear microgravity conditions showed increased resistance to general stress in exponential and stationary growth phases [50]. *Salmonella typhimurium* grown aboard the Space Shuttle mission STS-115 showed increased virulence under low shear microgravity conditions, compared to the same strain grown on Earth [51].

Aunins et al. (2018) studied the effect of microgravity on *E. coli* by analyzing the transcriptomic response of these bacteria grown on the ISS. Studied bacteria were grown in increasing gentamicin concentrations. In two days, *E. coli* grown in space, compared with the Earth control, adapted to higher gentamicin concentrations. Moreover, the strain on the ISS showed upregulation of 50 stress response genes, suggesting that microgravity induces stress responses associated with antibiotic stress and likely increases antibiotic tolerance in bacteria in space [3,11,52]. Indeed, in bacteria isolated from the ISS, increased resistance to antibiotics has often been reported. *E. faecalis* and *Staphylococcus* spp. isolates from the ISS have also been shown to harbor plasmid-encoded transfer genes, which facilitate the dissemination of antibiotic resistance. Therefore, these findings regarding ISS pathogens once again illustrates the necessity for novel, safe biofilm-combating or strongly biofilm-reducing approaches which can be easily used on the ISS and other spacecraft [3,11].

### 2.4. Biofilm Control Methods

**Figure 1:**
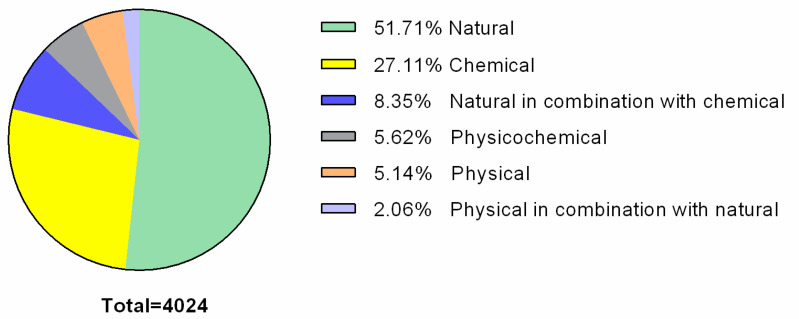
Summary of bacterial biofilm inhibition/control methods described during the last two years (2018–2020). PubMed total search results using “biofilm” as a keyword consisted of 15,315 entries, of which 4024 entries corresponded to articles regarding bacterial biofilm inhibition/control methods.

Over the last few decades, the search for and analysis of bacterial biofilm inhibition and control methods have begun to develop rapidly in order to discover the best option for their removal. To date, a wide range of research articles regarding bacterial biofilm control methods are available. Therefore, to summarize them, we performed an analysis of biofilm research performed during the last two years (August 2018–August 2020). To evaluate the prevalence of biofilm control/inhibition methods used for the past two years, the PubMed (NCBI) (https://www.ncbi.nlm.nih.gov/pubmed/) database was chosen for the analysis, as it is one of the most comprehensive. In order not to exclude search results that could be significant for the investigation, a general search term (keyword) “biofilm” was used. According to the scientific articles found in the PubMed database regarding biofilm inhibition and control, all methods, based on their nature, can be divided into six groups: (1) natural (biological) [53,54,55]; (2) chemical [56,57,58]; (3) physical [59,60]; (4) physicochemical [61,62,63]; (5) physical in combination with natural [64], and (6) natural in combination with chemical [65]. A summary of the bacterial biofilm inhibition/control methods described in the past two years is shown in Figure 1.

To date, only a few methods have been tested in order to find a suitable way to control microbial biofilm formation in space. In the majority of cases, these methods were tested on biofilms that were grown artificially in space conditions, subjected to real spaceflight conditions in a pre-formed state, or bacteria that could presumably form biofilms were isolated from areas such as potable water systems on spacecraft.

In 2010, Wong et al. tested disinfectants such as hydrogen peroxide (H_2_O_2_), colloidal silver, and buffered pH solutions on bacteria isolated from ISS water systems: *Ralstonia picketti*, *Burkholderia multivorans*, *Caulobacter vibrioides* and *Cupriavidus pauculus*. Results of the experiments showed that a single flush with either 6% H_2_O_2_ or a mixture of 3% H_2_O_2_ and 400 ppb colloidal silver solution effectively reduced bacterial concentrations for up to three months [9].

Perrin et al. (2018) performed a long-lasting VIABLE experiment (involving the evaluation and monitoring of microbial biofilms inside the International Space Station) that aimed to evaluate bacterial contamination on different spacecraft material surfaces [30]. These surfaces were subjected to varying pre-treatments with compounds such as biosurfactants, hydrogen peroxide, silica, and silver coating. Total bacterial load and the taxonomic composition of the microorganisms were evaluated by ATP-metry, qPCR, and 16S rRNA amplicon sequencing, respectively. The results of the study showed that the bacterial load on the studied surfaces was low and did not exceed the values allowed on the ISS. Taxonomic composition was detected to be similar to that found previously (*Enterobacteriales*, *Bacillales*, *Lactobacillales*, etc.) [30]. Finally, Perrin et al. (2018) concluded that no material or pre-treatment of the material used was shown to be better than any other, nor did it affect the quantity and taxonomy of the microbial contamination of ISS [30].

In 2018, Zea et al. began an extensive study aiming to design a spacecraft biofilm formation experiment with *P. aeruginosa* and *Penicillum rubens* as model microorganisms [3]. These microorganisms were planned to be cultured on different materials such as cellulose membrane, aluminium 6061, titanium Ti-6AL-4V, polycarbonate, quartz, silicon, stainless steel 316, carbon fiber, and lubricant-impregnated coupons using different nanotopographies to evaluate the changes in formed biofilm mass, thickness, morphology, and gene expression, and thus find the best antibacterial surfaces. However, so far, only the results of preliminary space-based testing to inform the spaceflight experiment design have been presented by the authors.

A more recent investigation revealed that bacteria such as *Cupriavidus metallidurans*, *Chryseobacterium gleum*, *Ralstonia insidiosa*, *R. pickettii*, *Methylorubrum (Methylobacterium) populi*, and *S. paucimobilis*, which were isolated from the ISS portable water system and presumably formed biofilms in spacecraft, are affected by bacteriophages and multiple predatory bacteria. The latter were tested in order to remove individual members of the biofilm in situ. However, the combination of precise and substantial depletion of a single target species was not achieved [10].

Sobisch et al. (2019) investigated the antimicrobial activity of AGXX^®^, a novel surface coating consisting of micro-galvanic elements of silver and ruthenium, along with examining the activity of a conventional silver coating. The antimicrobial materials were exposed on the ISS for 6, 12, and 19 months each, at a place frequently visited by the crew. The conventional Ag coating showed little antimicrobial activity. Microbial diversity increased with increasing exposure time on all three materials. After six months’ exposure on the ISS, no bacteria were recovered from AGXX^®^, after 12 months nine cultures were isolated, and after 19 months three cultures were isolated [4]. AGXX^®^ coating was previously invented and patented. This invention relates to the production and use of novel bioactive devices and metallic coatings, e.g., for sterilizing, disinfecting, and decontaminating water or aqueous solutions [66].

## 3. Antimicrobial Photoinactivation

**Figure 2:**
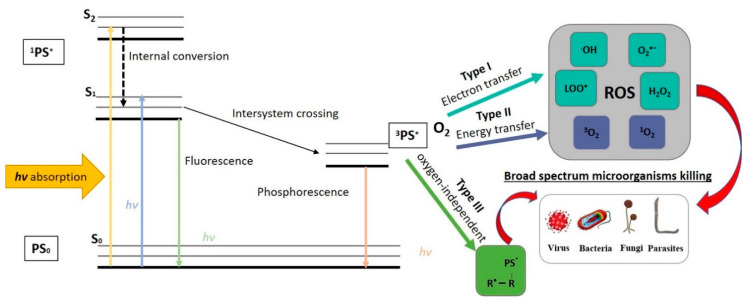
Schematic illustration of antimicrobial photoinactivation (API) mechanism (Jablonski diagram).

Antibiotic resistance by bacterial biofilms is an enormous problem in the world. Therefore, the development of alternative technologies could help to solve this problem. One of the promising approaches to controlling biofilms is the use of antimicrobial photoinactivation (API) (referred to as photodynamic therapy (PDT) when treating infectious disease or cancer). API is used not only to treat microbial infections but also to control the growth of microorganisms on various surfaces [67,68]. In this context, non-thermal and non-chemical API based on natural photosensitizers (PSs) might serve as a promising bacterial biofilm-and planktonic cell-decontamination tool in spacecraft. API is a modern biophotonic technology based on the interaction of a non-toxic PS, molecular oxygen, and low doses of harmless light of a suitable wavelength to match the PS absorption region [69]. Usually, the illuminated ground state (single-state) PS (1PS), located in the bacterial cells or at the bacterial cell surface, absorbs the light and is excited to its short-lived (*nsec*) singlet state, 1PS* (Figure 2). The excited-state electrons undergo intersystem crossing to a lower energy but longer-lived (µsec) PS triplet-state (3PS*) or return to the ground state by fluorescence emission and/or heat emission. The triplet-state PS interacts with molecular oxygen, electron donors or acceptors, and can produce reactive oxygen species (ROS). The different ROS generated by API mechanisms (upon photoinactivation) can migrate away from the formation site, destroy extracellular polysaccharides, and attack adjacent targets, including proteins, lipids, and nucleic acids present within the biofilm matrix, on the cell surface, and inside the microbial cells. Oxidation of sensitive biomacromolecules (proteins, DNA, RNA, lipids, etc.) initiates various forms of cellular damage and destroys bacterial cells in different growth modes (Figure 2) [70,71,72].

As shown in Figure 2, the photochemical reactions can be realized via a Type I or Type II mechanism and require proximity between the PS triplet state and the substrate. Both types of ROS can damage biomolecules and destroy or kill all known classes of pathogenic microorganisms. Type I reactions generate radicals following triplet state electron transfer from the PS triplet state to a substrate. A common terminal substrate for Type I reactions is molecular oxygen, leading to the production of radicals, such as superoxide anion (O_2_•‒), hydrogen peroxide (H_2_O_2_), hydroxyl radicals (OH•), carbonate radical anions (CO_3_•‒), and lipid-derived ions (LOO•), which oxidize biomolecules and cause cell damage and ultimately death [73]. In Type II mechanism reactions, the excited PS can transfer its energy directly to molecular oxygen (^3^O_2_) and form the highly reactive short-lived singlet oxygen (^1^O_2_*), which is a strong oxidant compared to ground state triplet oxygen [74]. Both types of reactions can occur simultaneously, but in most cases, API proceeds via a Type II reaction. All the same, the ratio between these processes still depends on the type of PS used and the microenvironment in which photosensitization is applied [75]. It is also important that the initially produced ^1^O_2_ can subsequently react with biological substrates (such as unsaturated fatty acids) to produce secondary radicals (such as lipid peroxide radicals) [76]. It allows the achievement of a significant decrease in the population of bacteria with minimal damage or thermal effects on the surrounding matrix [77,78]. Furthermore, recently, some scientists have proposed another oxygen-independent photoinactivation type, which they termed the “Type III photochemical pathway” [79]. This mechanism involves a photoinduced electron transfer that produces reactive inorganic radicals, which might be useful to inactivate various anaerobic bacteria.

It is important to note that one of the essential advantages of API compared to other antibacterial tools is the absence of any bacterial resistance to this treatment [74,80,81]. According to Liu et al. (2015), this is due to several reasons [74]:

(i) The time between administration of the PS and API is too short for the pathogen to develop resistance;
(ii) The PSs exhibit no dark toxicity (or very low toxicity), as a result of which bacteria do nothave to engage adaptive survival mechanisms against the PSs;
(iii) The cells are too damaged after API, disabling them to confer cross-generation additivity;
(iv) API does not target a single site in bacteria, so the ROS generated by this treatment target various pathogen cell structures and different metabolic pathways.

Numerous investigators have demonstrated significant reductions in both Gram-positive and Gram-negative bacteria (in vitro and in vivo) after API treatment [82,83,84,85]. The antibacterial efficiency of this treatment depends on many factors, including the physiological state of the bacteria, as well as their cellular structure and organization. Desirable properties of the PSs consist of optimal photophysical properties, pure chemical composition, acceptable stability and shelf life, water-solubility, no tendency to aggregate, high triplet state formation yield, low dark toxicity, resistance to photobleaching, high phototoxicity, small size for membrane permeation, minimal side effects, the ability to accumulate in bacteria or bind to the bacterial cell envelope, a broad-spectrum of antimicrobial action at relatively low concentrations, and a low light dose [86,87,88].

However, to date, there is no perfect PS that meets all the above mentioned characteristics. Most studied PSs are based on tetrapyrrolic macrocycles (porphyrins), chlorines, bacteriochlorins, phthalocyanines, and texaphyrins. These molecules have low toxicity, can form long-lived triplet excited states, and have high affinity to life-essential molecules. It has been found that PSs that fulfill these requirements should have a pronounced positive (cationic) charge [89]. It is known that a fundamental difference exists in the susceptibility to photosensitization between Gram-positive and Gram-negative bacteria. It is well documented that photosensitization-based inactivation is not effective enough to kill Gram-negative bacteria when neutral or anionic PSs are used. Neutral, anionic, or cationic PSs are mostly able to inactivate Gram-positive bacteria [90]. In contrast, Gram-negative bacteria are less susceptible to this treatment, due to their more conjugate cell wall structure and additional negatively charged outer membrane. They require a higher concentration of PS and light dose [91]. It is better to use cationic PSs or supplementation of API with permeabilizing agents to achieve a significant cell death of Gram-negative bacteria [90].

There are two main routes of PS–cell interaction. In the first case, it may form a stable conjugate with the surface of the cell wall. In this way, the PS is transported inside the cell, where it associates with the key structures and irreversibly damages them after photosensitization [80]. Demidova and Hamblin (2005) raised the hypothesis that there are three groups of antimicrobial PSs—(1) tightly bound, which penetrate microorganisms; (2) loosely bound; and (3) those which do not demonstrate binding [92].

**Figure 3:**
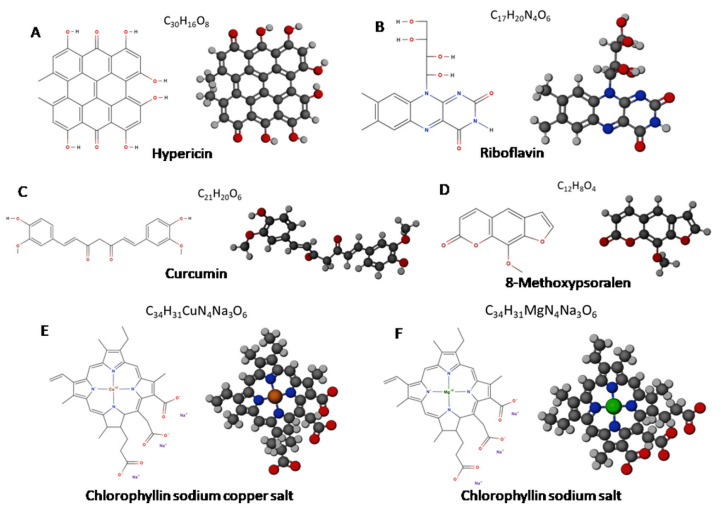
Chemical structure models of naturals photosensitizers (PSs) (2D and 3D).

PSs used for API are classified into four groups based on their structure and origin [19]: synthetic dyes, tetrapyrrole structures, natural PSs, and nanostructures. For bacteria biofilm inactivation in spacecraft, non-toxic, ecologically friendly, chemically pure, stable, non-bleaching, easy-to-produce, and water-soluble PSs should be used. Natural PSs meet the above-listed criteria and are one of the safest options of PSs for spacecraft use that have been proposed to date. Currently, four main natural products have been used for API: curcumin, riboflavin, perylenequinones (hypericin, hypocrellin), and psoralens [89]. Furthermore, chlorophyll derivatives have been used as natural PSs [93,94]. Moreover, several studies have recently reported that natural PSs extracted from plants can also be effective for the API approach [95,96] (see Table S1 in Supplementary Materials). Figure 3 depicts the chemical structures (2D and 3D models) of natural PSs used in API and discussed in this review.

Numerous API studies have demonstrated biofilm-eradication or substantial reduction, but few of them have used natural PSs. We thus review the recent literature concerning the efficiency of API based on natural PSs toward various bacterial species in biofilms, as well as planktonic growth modes. The review is mainly focused on biofilm-forming bacteria because this mode of growth not only poses a threat to spacecraft crew health but also damages spacecraft equipment and is difficult to eradicate from safety systems.

### 3.1. Perylenequinones

**Figure 4:**
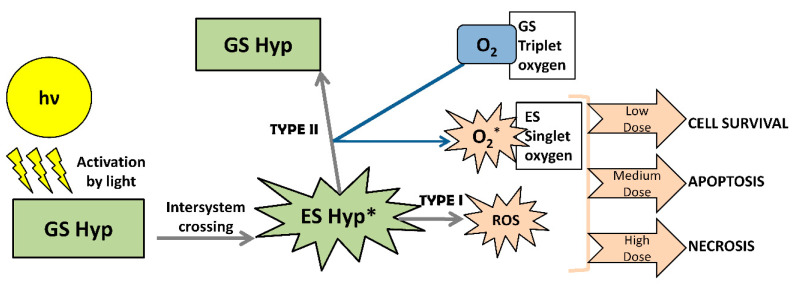
Schematic representation of the mechanism of Hyp photoactivation and induced damage; GS: ground state, ES: excited state.

The perylenequinones are a class of natural products characterized by a pentacyclic conjugated chromophore, giving rise to photoactivity. The most commonly used natural class B perylenequinone PS is hypericin (Hyp) (4,5,7,4′,5′,7′-hexahydroxy-2,2′-dimethylnaphtodiant hrone) (Figure 3A). Hyp is a naturally occurring red-colored anthraquinone derivative that comprises one of the main bioactive constituents of *Hypericum perforatum* (also called Saint John’s Wort). Hyp, which is almost insoluble in water, disperses in an aqueous physiological environment, producing non-fluorescent high-molecular-weight aggregates [97]. Therefore, it is soluble in 100% ethanol, methanol, or dimethyl sulfoxide (DMSO). Hyp exhibits an excitation maximum at 593 nm in DMSO solution and 544 nm in methanol [98]. As shown in Figure 4, upon light activation, at low concentrations, Hyp is efficient primarily in the generation of singlet oxygen (Type II mechanism), whereas, at high concentrations, it produces superoxide anion (Type I mechanism) [99].

Hyp (or its derivatives)-based API can induce death of Gram-positive methicillin-susceptible *S. aureus* (MSSA) and MRSA cells [100,101,102,103,104,105,106,107,108,109,110]. Hyp-mediated photoinactivation is reported to be more effective against planktonic *S. aureus* than against its biofilms [105,106,107]. The latter could be explained by the presence of polysaccharide intercellular adhesin (PIA) in the biofilm that blocks the uptake of hydrophobic Hyp. An excellent method to improve the effect of API is a chemical perturbation of the biofilm, for example, with acetylcysteine. Kashef et al. (2015) demonstrated that the combined use of Hyp and acetylcysteine increased the intracellular delivery of Hyp in *S. aureus* [107]. It has also been shown that Hyp-mediated API renders other Gram-positive bacteria including *Streptococcus mutans* and *Streptococcus sobrinus* [111], *L. monocytogenes* [112], *B. cereus* [113], *E. faecalis* [106] inactive.

It is known that Hyp is a neutral hydrophobic PS. Therefore, it does not simply get into Gram-negative bacteria such as *E. coli* [106,108,110,114,115], *P. aeruginosa* [100,106], and *Salmonella enterica* [112]. De Melo et al. (2013) used electroporation to increase the uptake of Hyp, thus enhancing the inactivation of *E. coli* to 3.5 logs [108]. Kairyte et al. (2012) combined Hyp-based API with high-power pulsed light treatment and increased the reduction of *S. enterica* from 1 to 6.7 log [112].

The fungal metabolites hypocrellin A (HA) and hypocrellin B (HB) are isolated from the parasitic fungi *Hypocrella bambusae* Sacc and *Shiraia bambusicola* P. Henn, respectively, which are found in different areas of Asia [88]. HB is known as a PS with high quantum yields of ^1^O_2_, low dark toxicity, a quick clearance rate, and availability in a pure monomeric form [116]. Jiang et al. (2013) used HB for the photoinactivation of Gram-positive *S. aureus* [117] and Gram-negative *E. coli* [118]. Otieno et al. (2020) found that 100 µM of HB in the presence of 72 J/cm^2^ light irradiation could obtain ~7 log reductions of *S. aureus, E. faecalis*, and *Streptococcus pneumoniae*. On the contrary, HB-based API efficacy on Gram-negative *E. coli* and *Klebsiella pneumoniae* were much lower. Gram-positive bacteria biofilms were also susceptible to API, but the efficiency was lower than that of planktonic cells [119]. Su et al. (2011) found that HA had API activity against Gram-positive *S. aureus* and *Bacillus subtilis*, and also against Gram-negative *E. coli* and *S.* Typhimurium when CaCl_2_ or MgCl_2_ was employed to weaken the permeability barrier [120].

In addition to the described bactericidal effects of Hyp [105,106], in vitro fungicidal [121,122] effects have also been reported. Light-activated Hyp is also considered to be an effective antiviral agent [123,124]. However, some clinical studies have revealed that high doses of Hyp can induce phototoxic skin reactions without showing any detectable antiviral or antiretroviral activity in patients with viral infections [125,126]. Hyp also possesses various positive or negative biological activities without light activation [127]. Therefore, for spacecraft biofilm decontamination applications, Hyp concentrations should be considered. Spacecraft surface API decontamination using Hyp could be potentially carried out, but for safety reasons, crewmembers’ exposure to it should be avoided during the process.

### 3.2. Riboflavin

Riboflavin (RF) (Figure 3B), also known as vitamin B_2_, is an essential micronutrient, which exhibits excellent photosensitive characteristics, plays a vital role in cell metabolism processes, and can be considered safe when administered to humans [128,129]. It is the main component of the cofactors flavin adenine dinucleotide (FAD) and flavin mononucleotide (FMN) in many flavoproteins and is ubiquitous among plants and animals [88,128]. RF is non-toxic and is labeled as “Generally Recognized As Safe” (GRAS) for consumption by the US Food and Drug Administration (FDA).

RF crystals have a yellow-orange color, whereas neutral solutions of RF have a green color. This is why they are used as food colorant known as E101. RF is one of the most widely studied compounds in terms of photostability and degradation in aqueous and organic solvents. RF in organic solvents shows strong absorption in the region of 270–271; 344–358 and 440–450 nm [130]. RF exhibits absorption maxima at 223, 267, 373, and 444 nm in the UV and visible regions in aqueous solution. It is degraded on exposure to light into various photoproducts: formylmethylflavin (FMF), lumichrome (LC), lumiflavin (LF), carboxymethylflavin (CMF), 2,3-butanedione, a β-keto acid, and a diketo compound [131]. The kinds of photoproducts formed by RF depend on the solvent, pH, buffer type, concentration, oxygen content, light intensity, and wavelengths.

**Figure 5:**
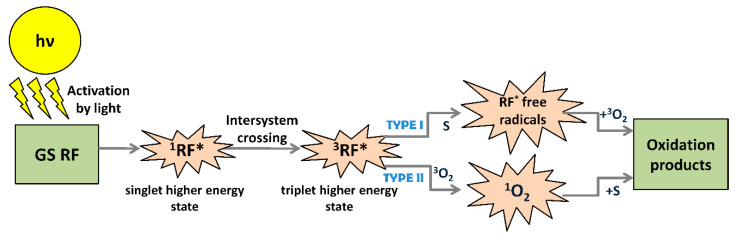
Photosensitized RF-mediated oxidation mechanisms. Upon activation by light, RF is excited to a singlet state of higher energy (1RF*), followed by intersystem crossing to an excited triplet state (3RF*). 3RF* can be involved in a photosensitized oxidation process of Type I or Type II. In the Type I mechanism, RF transfers the energy to a substrate (S) and generates RF* free radical species that interact with molecular oxygen in the ground state to yield oxidation products. In the Type II mechanism, RF transfers the energy to molecular oxygen in the ground state to generate the more reactive singlet molecular oxygen (^1^O_2_). The latter, in reaction with S, leads to the final oxidation products (according to [135]).

RF has been generally known to have antimicrobial properties. Therefore, light-induced activation of RF can selectively damage pathogens [132]. RF generates ROS such as superoxide radicals, hydroxyl radicals, hydrogen peroxide, and singlet oxygen when exposed to visible or UV light in the presence of oxygen [129,133]. The photooxidation of RF (Figure 5) includes either an electron transfer, which generates a superoxide ion and RF free radical species (Type I), or an energy transfer that causes the formation of singlet oxygen (Type II) [134]. The type I mechanism occurs preferentially at low oxygen concentrations [135].

It was found that RF, owing to its antimicrobial properties, can be applied to blood product sterilization and to a photocrosslinking procedure designed to stiffen the human cornea in patients with keratoconus [136,137]. Halili et al. (2016) found that 0.1% RF in the presence of 5.4 J/cm^2^ UV light (375 nm) irradiation could obtain a 98% reduction of Gram-positive *S. aureus* [138], whereas O’Rourke and Dowds (1992) showed the killing of *B. subtilis* [139]. However, Kashiwabuchi et al. (2012) failed to show any killing of *S. aureus* after RF-based API in the same treatment conditions [140]. A study by Makdoumi et al. (2010) resulted in the significant inactivation of various antibiotic-resistant bacteria (*Staphylococcus epidermidis*, *S. aureus*, *P. aeruginosa*, *E. faecalis*) mediated by RF photoactivated using UVA (365 nm) [141,142,143] and blue light (412 nm and 450 nm) [144,145] in thin layers of fluid (0.4–1.76 mm).

The antimicrobial efficacy of RF-based API was also presented by Maisch et al. (2014), who used cationic RF derivatives FLASH-01a and FLASH-07a for photoinactivation of *S. aureus*, *P. aeruginosa*, *E. coli*, and *Acinetobacter baumannii*. It was shown that the use of these PSs after light irradiation resulted in 6.6–6.7 log reductions in the viable counts of the bacteria [146].

Although RF belongs to the group of water-soluble vitamins, it is in fact one of the least soluble in water (0.10–0.13 g/L) [147]. FMN, which is produced from phosphorylation at the 50-position of the ribityl side-chain of RF, is also very sensitive to light. The water solubility of FMN is 200 times better than that of RF. Thakuri et al. (2011) obtained the killing of *S. aureus* and *P. aeruginosa* when FMN was excited by blue light [148], whereas Liang et al. (2015) also showed the significant killing of *E. coli* [149]. However, as was demonstrated by Wong et al. (2017), FMN photochemical treatment of *S. aureus* using a blue LED required a longer irradiation time than when using violet light [150].

### 3.3. Curcumin

Curcumin (CUR) is a natural yellow pigment extracted from the rhizomes of *Curcuma longa* that has been widely used as a foodstuff and as a spice. Since it is a foodstuff, it has attracted attention as a possible natural and non-toxic antimicrobial PS, particularly in dental applications. CUR is a relatively hydrophobic polyphenolic compound that is readily soluble in organic solvents (Figure 3C). It absorbs visible light in the 405–435-nm range, depending on the solvent [151]. Despite CUR’s widespread use as a PS in API, the mechanisms of its phototoxicity are not completely understood. Both Type I and II reactions are characteristic of CUR. It shows oxygen-dependent phototoxicity but is also known to be an antioxidant [152].

Several studies have verified its photoinactivation ability when activated with the proper wavelengths of light [88]. CUR-based API could induce significant killing of Gram-positive *E. faecalis* cells [153,154,155,156]. Pileggi et al. (2013) demonstrated that 10 µM of photoactivated CUR could completely inactivate *E. faecalis* biofilms after 30 min preincubation and 240-sec irradiation [156]. Meanwhile, Manoil et al. (2014) found that blue light-activated CUR can inactivate planktonic *S. mutans*, but the effect on biofilm was only 44% [157]. Paschoal et al. (2015) demonstrated that CUR could completely photoinactivate *S.mutans* by 5.97 logs when exposed to a blue LED light for 4 min (λ = 420 ± 20 nm) [158]. Therefore, a solution of CUR produced a 5.3-log reduction of a planktonic *S. mutans* suspension after 5 min irradiation with 450 nm light [159]. Araújo et al. (2017) showed that photoactivated CUR could kill cariogenic bacteria in vitro [160].

CUR-based API of Gram-negative bacteria is less studied. Penha et al. (2017) determined that photodynamic inactivation of Gram-negative bacteria (*E. coli*, *S. enterica*, *P. aeruginosa*) mediated by 75 μM CUR and 470 nm light was insignificant [161]. Haukvik et al. (2009) failed to show ~100% killing of *E. coli* after CUR-based API at 430 nm (light dose 30 J/cm^2^) in the same treatment conditions [155].

### 3.4. Psoralens

Psoralens are coumarins that possess a furan ring, which is why they are also called furanocoumarins (Figure 3D). Psoralens are commonly found in Rutaceae (*Ruta graveolens*) and Umbelliferaceae (*Apium graveolens*, *Angelica archangelic*, *Petroselenium crispum*) plants [162]. Psoralens are commercially derived from *Ammi majus*, a plant found in Egypt. Most psoralens have strong absorption bands in the range of 200–350 nm. Upon illumination with UVA/Vis light, psoralen molecules may undergo several reactions: Type I (electron transfer mechanism), Type II (electron-exchange type mechanism), Type III (photobinding to, e.g., DNA) [163]. The combination of 8-methoxypsoralen with UVA light (known as PUVA) was first introduced as a medical treatment for psoriasis [89]. PUVA has been used for the inactivation of bacteria, viruses, and protozoa in platelet and plasma blood components. However, now psoralens are not commonly used for human treatment, largely because of concerns over the long-term safety of ultraviolet light therapy and the toxic effects of psoralens administered orally [164]. Harding and Schwab (2012) used lime extract and synthetic psoralens to perform *E. coli* photoinactivation with UV radiation. It is believed that they may serve as enhancers of solar disinfection of water [165]. Because of the possible toxicity of psoralens, its usage for API in spacecraft for the decontamination of biofilms should be cautious, ensuring crewmembers are not exposed to this PS during the decontamination process.

### 3.5. Chlorophyllin

Although chlorophyll exhibits tetrapyrrole structures, as the most widespread phytochemical pigment in higher plants, algae and bacteria, it could also be relevant to the development of natural PSs. Chlorophyll and its derivatives have been identified as potential anti-mutagens and maybe chain-breaking antioxidants by acting as effective electron donors [166]. Chlorophyll derivatives exert strong photodynamic properties without the formation of toxic by-products [167].

Natural chlorophyll derivatives are widely used as PSs in photodynamic therapy. Various chlorophyll preparations are used in the industry as food colorants to reinforce or give a green color to manufactured products [168]. The chlorophyll molecule contains a cyclic tetrapyrrole nucleus with a coordinated magnesium atom at the center and a long hydrocarbon side chain attached through a carboxylic acid group [169]. Chlorophyll is a chlorin pigment, which is produced through the same metabolic pathway as other porphyrin pigments such as heme. Natural chlorophylls are so unstable that in most of the research, their semi-synthetic derivatives have been used as models for several experimental designs [170]. Chemical transformation of lipophilic chlorophylls into a freely water-soluble sodium salt derivative involves the removal of the phyttyl tail and the additional replacement of the central coordinated Mg^2+^ with Cu^2+^ (Figure 3E) [171]. The first type of these compounds is a sample called sodium chlorophyllin (Chl), whereas the second one is known as chlorophyllin sodium copper salt (CuChlNa). Chlorophyllins can be simply extracted from different plant sources such as spinach, grass, dandelion, green cabbage, water hyacinth, and algae [94,172]. CuChlNa is a water-soluble food colorant (E-140ii) and is widely used in dietary supplements and cosmetics [173,174].

Chl is obtained by saponification of the solvent-extracted products from edible plant material, grass, lucerne, and nettles [168]. It contains water-soluble chlorophyll derivatives and is marketed as a grey-green powder after dehydration of the chemical preparation [174]. It should be noted that Chl is efficient primarily in the generation of singlet oxygen (Type II mechanism). Moreover, the expression of genes (*OxyR*, *AhpC*, *GrxA*, *SulA*, *AtpC*, *groEL*, *STM0225*) after API confirmed that treated cells of bacteria survived oxidative stress induced by emerging ROS. Eventually, it was found that API induces significant cell membrane disintegration, which enables extensive leakage of DNA and protein components and causes shrinkage of cells [175].

Chl preferentially associates with microorganisms and, after activation with visible light of an appropriate wavelength, generates ROS, which induce lethal damage of bacteria. Several studies have verified Chl’s photodynamic killing ability of Gram-positive bacteria when PS-activated with the proper wavelengths of light. Kreitner et al. (2001) studied inactivation of agar-plated Gram-positive *S. aureus*, *B. cereus*, and *B. subtilis* by 10^–5^ M Chl-based API [176]. Luksiene et al. (2013, 2010, 2011) used 10^−8^–10^−7^ M Chl and 405 nm light and achieved complete photoinactivation of Gram-positive *L. monocytogenes* and *B. cereus* in phosphate buffered saline (PBS) buffer [177,178,179]. However, the Chl-based API effect is lower against Gram-negative bacteria such as *E. coli* and *S. enterica* [175,177,180,181]. To increase the susceptibility of Gram-negative bacteria to Chl-based API, Chl-chitosan conjugate [175,181], high power pulsed light [175], porphyrin precursor ALA [177], or lower dose antibiotics could be used [182].

To conclude this Section, most of the currently accepted API methods are applied in the medical area. In recent decades, API was used for the treatment of various microbial infections and to control the growth of microorganisms on various surfaces [80,183]. Several papers have also been published on the photodynamic inactivation of microorganisms regarding food surface decontamination. API can be used for cleaning and sanitation of food-processing and food-handling surfaces as well [80]. Berg et al. (2014) patented a microbial growth controlling method based on surface treatment with one or more photosensitizers and further exposure to electromagnetic radiation [67]. Luksiene and Buchovec (2009) patented a method of food and food-related surface decontamination using non-thermal irradiation and food-friendly PSs [68]. The effective photoinactivation of different physiological forms of bacteria obtained in vitro and on various surfaces looks promising and may serve as a background for the further development of a novel, safe, hurdle technology for the decontamination of confined spacecraft systems. In food and medical settings PSs are usually sprayed, spread, and infused onto surfaces, or sometimes incorporated into them [184,185]. For spacecraft biofilm decontamination applications, PSs can be applied analogically—by spraying, spreading, and/or infusing them onto surfaces, as is done on Earth. Nevertheless, in order to enable safe and effective API applications in spacecraft, suitable concentrations of PSs and specific light doses must be optimized. Optimal API conditions should be studied on Earth in advance in simulated space conditions.

## 4. Illumination Requirements for API

**Figure 6:**
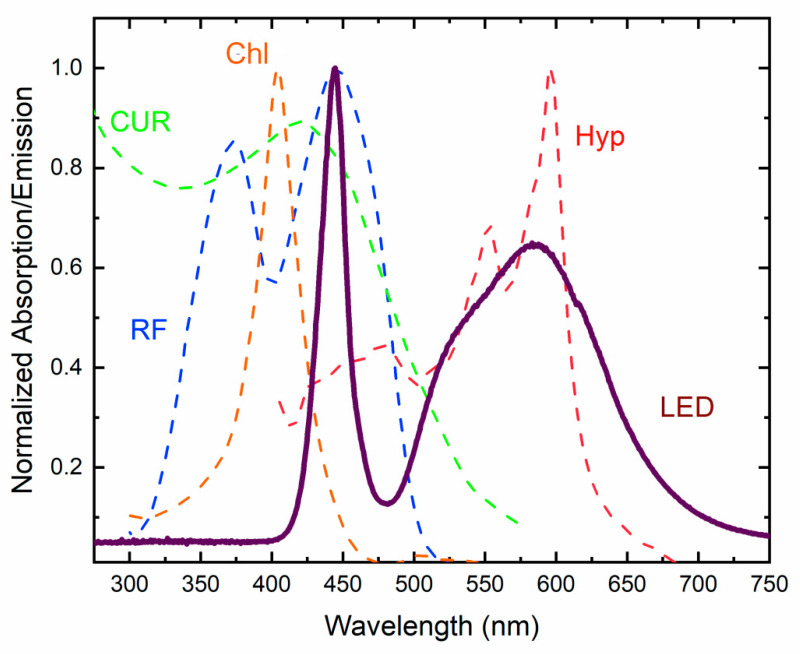
Absorption spectra of CUR [160], RF, sodium Chl, Hyp [113], and typical emission spectra of an LED (4000 K).

An indispensable part of API and PDT is proper illumination. The operational mechanism of API requires the presence of light in order to excite internal electrons into excited states (Figure 2). In particular, a proper spectrum and sufficient amount of excitation (dose) have to be provided for the desirable inactivation effect. In general, one has to illuminate the PSs with light of a photon-energy greater or equal to the energy distance between the ground state PS_0_ and excited state 1PS*. Still, the absorption curves of certain PSs are the most accurate characteristics to follow (Figure 6).

We can see that most of the PSs require high-photon-energy light of a blue color or even UV light. For the application of PSs for sterilization purposes on Earth or in spacecraft, the excitation by general illumination systems would be desirable. Any dedicated illumination/treatment system results in extra cost and weight, which is crucial for spacecraft. PSs such as CUR and RF show a good overlap with the “blue” part of white LED light, whereas Hyp can be excited by the phosphor-converted yellow-green light. On the other hand, PSs such as Chl and others requiring light of <420 nm remain beyond the spectrum of modern (LED-based) general illumination spectra, requiring the implementation of dedicated illumination/treatment sources emitting light in the violet–UV spectral range.

Furthermore, the required illumination doses have to be discussed and compared with general illumination conditions. RF can be excited by blue light and requires from 3 to 400 J/cm^2^ according to the data available in the literature (see Table S1 of Supplementary Material) [93,94,95,96,100,101,102,103,104,105,106,107,108,109,110,111,112,113,114,115,117,118,119,120,138,139,140,141,142,143,144,145,146,148,149,150,153,154,155,156,157,158,159,160,161,165,173,175,176,177,178,179,180,181,182,186,187,188,189,190,191,192,193,194,195,196,197,198,199,200,201]. Typical illumination of a work plane according to the international norms is from 300 to 500 lx and does not exceed 1000 lx (except surgery). If typical LEDs of 4000K are used (see Figure 6), (320 lm/W, the luminous efficacy of radiation), the surface irradiation ranges from 0.9 to 1.56 W/m^2^ of white light spectrum (400–700 nm). Therefore, we have only 20 to 35 mW/cm^2^ of blue light irradiance (~22% of white). Illumination for 24h provides an illumination dose of 1.73 to 3.01 J/cm^2^. For Hyp, accepting a wider range of excitation, about two times’ better overlap is observed, resulting in 3.5 to 6 J/cm^2^ per day. Such doses can be a starting pointing for photoinactivation but are not always sufficient. Therefore, we have to conclude that the implementation of photoinactivation techniques for disinfection with the employment of general artificial lighting requires further study and scientific discussion since it is not clear if the photoinactivation dynamics measured for relatively short treatment times (up to a few hours) could be validated for a few days or weeks.

## 5. Conclusions

Biofilms play an important role in human infections and pose a potential threat to material integrity, not only in confined facilities such as hospital and food settings on Earth but also, in spacecraft. The dominant organisms are mostly associated with the microbiome of crewmembers’ skin and largely contribute to both planktonic and biofilm microbial diversity of the spacecraft. Some of the bacteria may include opportunistic pathogens as well. According to the latest research on bacterial biofilm development in space conditions, biofilms are currently considered to pose a more significant threat as destroyers of abiotic surfaces of various forms of spacecraft equipment, rather than posing a health threat to the crewmembers. Nevertheless, some studies revealed that in spaceflight conditions, biofilms form faster and, therefore, can acquire antibiotic resistance faster, forming thicker structures and assuming different forms in space. Therefore, studies of longer duration are needed in order to correctly evaluate the possible effect of bacteria in space conditions on crewmembers’ health and to investigate how their space-altered immune systems are able to respond to infections.

This review has also focused on the recent scientific literature concerning the efficiency of antimicrobial photoinactivation based on natural PSs (perylenequinones, riboflavin, curcumin, psoralens, and chlorophyllin) toward various bacterial species in planktonic and biofilm growth modes. It was found that a significant number of natural products and their derivatives have shown proven photodynamic antibacterial actions in confined on-ground settings, such as hospitals, and are also used in food protection. Therefore, API potentially could be applied in a closed spacecraft environment as a safe and non-toxic method, to which bacteria do not develop resistance. Nevertheless, the experimental data on biofilm control in the spacecraft environment, especially concerning natural antibacterial techniques, is currently episodic and requires more attention and scientific interest. Furthermore, the incorporation of practical illumination systems into spacecraft lighting is still somewhat obscure, and also requires further study.

## Supplementary Materials

Supplementary Materials can be found at https://www.mdpi.com/1422-0067/21/18/6932/s1.

## Abbreviations

ISS	International Space Station
API	Antimicrobial photoinactivation
PS	Photosensitizer
ROS	Reactive oxygen species
CUR	Curcumin
RF	Riboflavin
Hyp	Hypericin
Chl	Chlorophyllin
EPS	Extracellular polymeric substances
LSMMG	Low-shear modeled microgravity
MRSA	Methicillin-resistant *S. aureus*
PWD	Portable water dispenser
TSB	Tryptic soy broth
PDT	Photodynamic therapy
DMSO	Dimethyl sulfoxyde
GS	Ground state
ES	Excited state
MSSA	Methicillin-susceptible *S. aureus*
PIA	Polysaccharide intercellular adhesin
HA	Hypocrellin A
HB	Hypocrellin B
FAD	Flavine adenine dinucleotide
FMN	Flavine mononucleotide
GRAS	Generally regarded as safe
FDA	Food and Drug Administration
FMF	Formylmethylflavine
LC	Lemichrome
LF	Lemiflavin
CMF	Carboxymethylflavin
PBS	Phosphate buffered saline
