# Supplementary material for: Antimicrobial Photoinactivation Approach Based on Natural Agents for Control of Bacteria Biofilms in Spacecraft
Source: Int J Mol Sci. 2020 Sep 21;21(18):6932. doi: 10.3390/ijms21186932 (PMC7554952; doi:10.3390/ijms21186932)
Supplement: Supplementary file 1 [file ijms-21-06932-s001.pdf]

Table 1. Review of natural PSs-based API conditions used in different studies.

Ref.	Photosensitizer (PS)	Bacteria species/strains (CFU×mL^{-1})		Modes of growth	Concentration of PS/dark toxicity	Preincubation time/medium	Light source; wavelength; irradiance	Light dose/illumination time	API efficiency
106	Hypericin (Hyp)	*Staphylococcus aureus* (10^6–10^7)	ATCC 25923	[1]P	1 µg/mL/ [3]DS	5 min/ [4]PBS buffer	[5]LED; 590 nm; 80 mW/cm^2	48 J/cm^2/10 min	6.3 log_{10}
		Escherichia coli (10^6–10^7)	ATCC 25922						6.2 log_{10}
		Enterococcus faecalis (10^6–10^7)	ATCC 1700						6.5 log_{10}
		Pseudomonas aeruginosa (10^6–10^7)	ATCC 27853						0.7 log_{10}
107	Hyp	*S. aureus* ($6×10^8$)	ATCC 25923	P	0.5 µg/mL/DS	5 min/PBS buffer	LED; 590 nm; [6]—	8 J/cm^2/5 min	6.5 log_{10}
				[2]B				16 J/cm^2/10 min	[7]NS
			UTMC 1484	P				8 J/cm^2/5 min	5.5-6.7 log_{10}
				B				16 J/cm^2/10 min	NS
			UTMC 1474	P				8 J/cm^2/5 min	5.5-6.7 log_{10}
				B				16 J/cm^2/10 min	NS
			all type	B	0.5 µg/mL/DS	10 min/ acetylcysteine			5.2-6.3 log_{10}
100	Hyp	*P. aeruginosa* (10^7–10^8)	PAO1	P	10 µM/DS;	30 min/ [8]LB medium	LED; 590 nm; 150 ±20 W/m^2	162 J/cm^2/180 min	NS
		S. aureus (10^7–10^8)	KCTC 3881					54 J/cm^2/60 min	~ 6.5 log_{10}
			KCTC 1916						~ 7 log_{10}

			2659 (MRSA)						~ 5.5 log$_{10}$
105	Hyp	*S. aureus* (10^6)	ATCC 29213 (MSSA)	P	0.25 µM/DS	5 min/ [9]TSB medium	LED; 602±10 nm; 14 mW/cm^2	8 J/cm^2/10 min	complete (6 log$_{10}$)
				P	0.015 µM/DS			25 J/cm^2/30 min	complete (6 log$_{10}$)
				B	0.015 µM/DS	24 h/PBS buffer		25 J/cm^2/30 min	3.5 log$_{10}$
			ATCC 33591 (MRSA)	P	1 µM/DS	5 min/TSB medium		8 J/cm^2/10 min	complete (6 log$_{10}$)
				P	0.015 µM/DS			25 J/cm^2/30 min	complete (6 log$_{10}$)
				B	250 µM/DS	20 min/PBS buffer		25 J/cm^2/30 min	2.3 log$_{10}$
112	Hyp	*Salmonella enterica* (10^7)	DS88(SL5676 Smr Plm32)	P	$1{\times}10^{-5}$ M/DS	60 min/PBS buffer	LED; 585 nm; 3.84 mW/cm^2	13.8 J/cm^2/–	1 log$_{10}$
							LED+HPPL	13.8 J/cm^2 + 0.023 J/cm^2/–	6.7 log$_{10}$
		Listeria monocytogenes (10^7)	ATC$_{L3}$C 7644		$1{\times}10^{-7}$ M/DS	60 min/PBS buffer	LED; 585 nm; 3.84 mW/cm^2	9.22 J/cm^2/–	7 log$_{10}$
						5 min/PBS buffer		2.3 J/cm^2/–	2.2 log$_{10}$

							LED+HPPL	2.3J/cm² + 0.023 J/cm²/—	7 $\log_{10}$
113	Hyp	*Bacillus cereus* (10^7)	ATCC 12826	P	1×10^{-7} M/DS	2 min/PBS buffer	LED; 585 nm; 3.84 mW/cm²	9.2 J/cm²/40 min	3.5 $\log_{10}$
						60 min/PBS buffer			4.4 $\log_{10}$
					1×10^{-7} M/DS	2 min/PBS buffer			2.2 $\log_{10}$
						60 min/PBS buffer			2.5 $\log_{10}$
115	Hyp	*E. coli* (10^7)	—	P	36 µM/DS	68 min/PBS buffer	LED; 590 nm; 16.4 mW/cm²	5.9 J/cm²/260 sec	4.1 $\log_{10}$
102	Hyp	*S. aureus* (~ 10^7)	CECT 239	P	4 µM; washed/DS	30 min/PBS buffer	LED; green light; —	18 J/cm²/15 min	~ 7 $\log_{10}$
						30 min/20% PBS-DMSO mixture			~ 6.5 $\log_{10}$
					4 µM; washed/DS	30 min/PBS buffer			~ 6 $\log_{10}$

						30 min/20% PBS-DMSO mixture			~ 5.8 log_{10}
	Hyp-2ß-LG (Hyp complex with the dimeric form of β-lactoglobulin				4 µM-200 µM/DS	30 min/PBS buffer		18 J/cm²/5 min	5 log_{10}
						30 min/20% PBS-DMSO mixture			~ 3 log_{10}
					4 µM -200 µM; washed/DS	30 min; PBS buffer			~ 5.8 log_{10}
						30 min/20% PBS-DMSO mixture			~ 3 log_{10}
109	Hyp	*S. aureus* (~ 10^7)	CECT 239	P	8 µM/DS	30 min; PBS buffer	LED; green light; ⊢⊣	18 J/cm²/15 min	~ 7 log_{10}
	Hyp-2ß-LG				8 µM -10 µM/DS			37 J/cm²/30 min	5 log_{10}
101	Hyp	*S. aureus* (~ 10^7)	CECT 239	P	10 µM/DS	30 min/PBS buffer	LED; green light; ⊢⊣	18 J/cm²/15 min	~ 7 log_{10}
	Hyp-ApoMb (Hyp and apomyoglobin complex)				10 µM (1:2)/DS			37 J/cm²/30 min	~ 5.5 log_{10}
104	PVP-Hyp (synthesized by non-covalently binding Hyp to polyvinylpyrrolidone)	*S. aureus* (~ 10^8)	DSMZ 18587	P	100 nM/DS	5 min/TSB medium	1200 red light halogen lamp; 75 mW/cm²	135 J/cm²/30 min	5 log_{10}

110	Hyp	*S. aureus* (10^8)	Clinical isolate (MRSA)	P	8 µM/DS	30 min/PBS buffer and[10]NB medium	400 W quartz-halogen lamp; 600-800 nm; 14 mW/cm^2	30 J/cm^2/ —	complete (> 6 log$_{10}$)
			ATCC 25923 (MSSA)						
		E. coli (10^8)	Clinical isolate (ESBL-producing)		40 µM/DS				0.18 log$_{10}$
			ATCC 25922						
108	Hyp	*E. coli* (10^8)	ATCC 25922	P	4 µM/DS	10 min; PBS buffer	Lumacare LC122 lamp; 590 nm; 100 mW/cm^2	40 J/cm^2/—	0.93 log$_{10}$
		S. aureus (10^8)	ATCC 35556						4.25 log$_{10}$
	Hyp	*E. coli* (10^8)	ATCC 25922				Hyp (Light)+ EP (2 electric pulses at 1000 V/cm, 50 µs long, 1Hz)	40 J/cm^2/—	3.5 log$_{10}$
		S. aureus (10^8)	ATCC 35556						6.85 log$_{10}$
111	Hyp	*S. mutans* (10^8)	OMZ 918	P	10 µg/mL/DS	30 min/ [11]FUM medium	halogen polymerization device; 400-505 nm; 1070 mW/cm^2	256.8 J/cm^2/2×120 s	99.9 %
		S. sobrinus (2.16×10^8)	OMZ 176		2.5 µg/mL/DS	15 min/FUM medium		128.4 J/cm^2/120 s	100%
114	Hyp	*Shigellaflexneri*(10^9)	1a	P	2.4×10^{-5} M/dm^3/Toxic effect	NaCl/dimethyllformamide	white light; 5mW/cm^2	27 J/cm^2/90 min	1.3%
			1b						78.3 %
			2a						37.2 %

		E. coli (10^9)	K12 781						1.2 %
			K1 959						47.6 %
		B. subtilis(10^9)	3						24.3 %
103	aHyp (non-purified, hydrophilic extract of *Hypericumperforatum*)	*S. aureus* (~ 10^7)	ATCC 25923	P	1 µM/DS	30 min; PBS buffer	LED; 515 nm; 16 mW/cm^2	9.6 J/cm^2/10 min	7 log_{10}
117	Hypocrellin B	*S. aureus*(10^8)	–	P	2.5 µM/DS	60 min/–	LED; 470 nm; 60 mW/cm^2	0.3 J/cm^2/–	~ 1.8 log_{10}
118	Hypocrellin B	*E. coli*(10^8)	DH5α	P	25 µM/DS	60 min/LBmedium	LED; 470 nm; 60 mW/cm^2	12 J/cm^2/–	~ 90 %
119	Hypocrellin B	*S. aureus*(10^7)	ATCC 29213	P	100 µM/DS	30 min/PBS buffer	50 W xenon lamp; 400-780 nm; 0.08 W/cm^2	72 J/cm^2/900 sec	~ 7 log_{10}
				B					3.56 log_{10}
			Clinical isolate (MRSA)	P					~ 7 log_{10}
				B					3.71 log_{10}
		E. faecalis(10^7)	ATCC 29212	P					~ 7 log_{10}
				B					3.29 log_{10}
			VRE (ATCC 51299)	P					~ 7 log_{10}
				B					3.35 log_{10}

		S. pneumonia (10^7)	ATCC 49619	P					~ 7 $\log_{10}$
				B					3.15 $\log_{10}$
			Clinical isolate of MDR	P					~ 7 $\log_{10}$
				B					3.36 $\log_{10}$
		E. coli(10^7)	ATCC 25922	P					2 $\log_{10}$
		K. pneumonia (10^7)	ATCC 700603	P					2 $\log_{10}$
120	Hypocrellin A	*S. aureus*(10^7-10^8)	6538	P	1 μM/DS	60 min/PBS buffer	50W halogen–tungsten lamp; 30 mW/cm^2	126 J/cm^2/120 min	The survival 0.02%
		B. subtilis(10^7–10^8)	9372			60 min/PBS buffer			The survival less than 0.1%
		E. coli(10^7–10^8)	ATCC 25922			60 min/PBS buffer			NS
						+$CaCl_2$			decreased the survival to 1.2%
						+$MgCl_2$			decreased the survival to 1.7%
		Salmonella Typhimurium (10^7–10^8)	50013			60 min/PBS buffer			NS
187		*in situ* oral biofilms		B	32 mg/mL/–			60 J/cm^2/5 min	complete

	Hypericumperforatum extract					2 min; intraoral devices with fixed bovine enamel slabs	VIS + wIRA; 200 mW/cm^2		
138	Riboflavin (RF)	*S. aureus* (10^5)	MRSA Strain 1	P	0.1%/11% inhibition	0 min/NB agar	LED; 375 nm; 3 mW/cm^2	5.4 J/cm^2/30 min	98%
							ambient light (300 lux); 107 µW/cm^2	0.193 J/cm^2/30 min	13%
			MRSA Strain 2	P	0.1%/3% inhibition		LED; 375 nm; 3 mW/cm^2	5.4 J/cm^2/30 min	100%
							ambient light (300 lux); 107 µW/cm^2	0.193 J/cm^2/30 min	15%
188	RF	*L. monocytogenes*; 4 strains cocktail (3.5 log CFU/cm^2)	1/2a	P	100 µM/DS	–/on smoked salmon	LED; 460 nm; 15 mW/cm^2	2400 J/cm^2/–	1.2 log CFU/cm^2 (at 4 ºC)
			1/2b						
			3a						
			4b						1.1 log CFU/cm^2 (at 12 ºC)
189	RF	*E. coli* (10^7)		P	50 µM/DS	0 min/PBS buffer	Visible white light; 38.6 W/m^2	27,8 J/cm^2/2 h	~ 4.5 log$_{10}$
145	RF	*S. aureus* (8×10^5)	MRSA	P	0.01%/DS	0 min/fluid layer of 1.2 mm	450 nm; –	84 J/cm^2/–	complete

190	RF	*S. epidermidis*(6.5×10⁷)	ATCC 12228	P	50 µM/DS	–/in platelet concentrates	UV lamps; 265 – 370 nm; –	6.2 J/ cm²/8-10 min	≥ 4.15 log
		E. coli (1.1×10⁸)	ATCC 25922						≥ 4.38 log
142	RF	*S. epidermidis* (1.6–2.5×10⁴)	RS 30 (s1) ATCC 12228	P	400 µM/DS	–/in thin layers of fluid (0.4–1.76 mm)/RPMI (cell culture media)	UV-X;365nm; 2.7-3.3 mW/cm²	10.8 J/cm²/60 min	mean reduction from start point ranging between 86 and 90%
		S. aureus (2.5–4.0×10⁴)	RS 12(s1) CCUG 15915						mean reduction from start point ranging between 61 and 71%
			RS (s1) ATCC 29213						
		P. aeruginosa (9.4×10³ –3.1×10⁴)	RS 11(s1) CCUG 17619						mean reduction from start point ranging between 98 and 100%
			RS 11 (s1) ATCC 27853						
141	RF	*S. epidermidis* (1.7–3.37×10⁴)	ATCC 12228	P	0.03%/DS	–/in thin layers of fluid (0.4–1.76 mm); RPMI (cell culture media)	UVA; 365 nm; –	10.8 J/cm²/60 min	80%
					0.09%/DS				50%

143	RF	*S. epidermidis* (1.7–3.37×10^4)	ATCC 12228 (S)	P	0.01%/DS	−/in thin layers of fluid (0.4–1.76 mm); RPMI (cell culture media)	UVA; 365nm; 3 mW/cm^2	5.4 J/cm^2/30 min	80%-92%
			CCUG35257 (methicillin and oxacillin R)						
		S. aureus (1.7–3.37×10^4)	ATCC29213/CCUG15915(S)						60%-70%
			ATCC43300/CCUG41586 (oxacillin R, MecA+, nuc+)						80%-92%
		P. aeruginosa (1.7–3.37×10^4)	ATCC27853(S)						80%-92%
			CCUG37384 (ciprofloxacin R)						80%-92%
		E. faecalis (1.7–3.37×10^4)	ATCC29212/ CCUG9997 (S)						60%-70%
			CCUG34062 (vancomycin R)						

144	RF	*S. aureus* (1.7–3.37×10^4)	CCUG41586/ATCC 43300 (MRSA)	P	0.01%/DS	—/RPMI (cell culture media)	commercially available lamp for treatment of acne TRIA Beauty lamp; 412 nm; 47.7 mW/cm^2	28.6 J/cm^2/10 min	99%
							prototype lamp; 450nm; 47.4 mW/cm^2	28.4 J/cm^2/10 min	98%
139	RF	*B. subtilis* (—)	—	P	10 µM/DS	10 min in glucose minimal medium	**500** watt tungsten-halogen lamp; —	—/20 min	effective
140	RF	*S. aureus* (~ 10^8)	ATCC 29213	P	0.1 %/DS	0 min/NB medium	UVA;365 nm; 3 mW/cm^2	5.4 J/cm^2/30 min	Not susceptible
146	FLASH-01a (synthesized RF derivative with	*P. aeruginosa* (10^8)	ATCC BAA-44 (MRSA)		100 µM/DS	10 s/aqueous solutions	Non-coherent light; 380-600 nm;	1.5 J/cm^2/30 s	6.8 log_{10}

	single positive charge)	*E. coli* (10^8)	O104:H4 (EHEC)		50 µM/DS;		50 mW/cm^2	12 J/cm^2/240 s	6.5 log_{10}
		Acinetobacterbaumannii (10^8)	clinical isolate		100 µM;/DS			9 J/cm^2/180 s	6.6 log_{10}
		S. aureus (10^8)	ATCC 25923 (MRSA)		50 µM/DS			3 J/cm^2/60 s	6.6 log_{10}
	FLASH-07a (synthesized RF derivative with eight positive charge)	*P. aeruginosa* (10^8)	ATCC BAA-44 (MRSA)		50 µM/DS			3 J/cm^2/60 s	6.7 log_{10}
		E. coli (10^8)	O104:H4 (EHEC)		50 µM/DS			9 J/cm^2/180 s	6.6 log_{10}
		A. baumannii (10^8)	clinical isolate		50 µM/DS			4.5 J/cm^2/90 s	6.7 log_{10}
		S. aureus (10^8)	ATCC 25923 (MRSA)		100 µM/DS			1.5 J/cm^2/30 s	6.5 log_{10}
148	FMN (RF-5-phosphate)	*S. aureus* ($1.5{\times}10^8$)	clinical isolate	P	1 mg/dl/DS	15 min/PBS buffer	LED; blue light; –	–/120 min	~ 4.5 log_{10}
		P. aeruginosa ($1.5{\times}10^8$)						–/180 min	~ 6 log_{10}
149	FMN	*E. coli* ($1.2{\times}10^{10}$)	DH5α	P	30 µM/DS	0 min/PBS buffer	LED; 462 (410–510) nm; 2 mW/cm^2	14.4 J/cm^2/120 min	96%
150	FMN	*S. aureus* (10^7)	BCRC 10451	P	120 µM/DS	0 min/PBS buffer	Blue light; 2 mW/cm^2	14.4 J/cm^2/120 min	94.9 %
					30 µM/DS		Violet light; 1 mW/cm^2	1.8 J/cm^2/30 min	96.3 %

			MRSA JD004		120 μM/DS		Blue light; 2 mW/cm²	14.4 J/cm²/120 min	95.2 %
191	Curcumin (CUR)	*L. monocytogenes*(10^8)four strain cocktail	ATCC 19115<br>ATCC 13932	P	0.5 μM/DS	20 min/0.85% physiological saline	LED; 455-460 nm; 1.8 mW/cm²	1.62 J/cm²/15 min	complete (7.65 log_{10})
			ATCC 7644<br>4bLM	B	20 μM/DS			6.48 J/cm²/60 min	OD_{600}: from 2.10 to 0.93
192	CUR	Intact dentin caries biofilms	3-day B		600 μM/L; 45 % reduction after 5 min incubation	1min/ Glass slabs	LED; 455 nm; 40 mW/cm²	75 J/cm²/–	62%
						5 min/ Glass slabs			~ 62 %
			5-day B		600 μM/L; ~ 22% reduction after 2 min incubation	1 min/ Glass slabs			17%
						5 min/ Glass slabs			~ 50 %
160	CUR	*S. mutans* and *Lactobacillus acidophilus* (grown simultaneously in dentine carious lesions (10^8)			5 g/L/DS	to inoculate the bacterial-induced caries on human dentine slabs	LED; 450 nm; –	5.7 J/cm²/5 min	~ 70%
								5.7 J/cm²/2 min	
193	CUR	*Propionibacterium acnes* ($2×10^5$)	BCRC 10723	P	25 μM/ inhibition rate below 20 %	–/RCM medium	LED; 462 nm 3 mW/cm²	0.09 J/cm²/30 sek	100%
153	CUR	*E. faecalis* ($5×10^9$)		P	136 μM/DS	5 min;TSBmedium	LED;	12.5 J/cm²	

			ATCC 29212				450 nm; 151 mW/cm^2		completely eliminate
		P. acnes (5×10^9)	ATCC 6919		14 µM/DS	5 min/BHI medium			
161	CUR	*S. aureus* (10^7)	ATCC 25923	P	75 µM/DS	10 min/0.85% saline	LED; 470 nm; ━	139 J/cm^2/10 min	3.27 log_{10}
								278 J/cm^2/20 min	~ 3.57 log_{10}
								417 J/cm^2/30 min	complete (~ 6 log_{10})
		Aeromonashydrophilia (10^7)	ATCC 7966					139 J/cm^2/10 min	3.33 log_{10}
								278 J/cm^2/20 min	complete (~ 6 log_{10})
								417 J/cm^2/30 min	complete (~ 6 log_{10})
		E. coli (10^7)	ATCC 25922					139 J/cm^2/10 min	1.29 log_{10}
								278 J/cm^2/20 min	2.65 log_{10}
								417 J/cm^2/30 min	complete (~ 6 log_{10})
		S. Typhimurium (10^7)	ATCC 14028					139 J/cm^2/10 min	1.26 log_{10}
								278 J/cm^2/20 min	1.81 log_{10}
								417 J/cm^2/30 min	2.82 log_{10}
		P. aeruginosa (10^7)	ATCC 27853					139 J/cm^2/10 min	0.24 log_{10}
								278 J/cm^2/20 min	0.3 log_{10}
								417 J/cm^2/30 min	0.33 log_{10}
194	CUR	*S. aureus* (10^8)		P	2.5 µM/DS	60 min/LB medium	LED; 470 nm; 60 mW/cm^2	3 J/cm^2/━	2 log_{10}

195	CUR	*S. aureus* (10^6)	ATCC 25923 (MSSA)	P	5 µM/DS	20 min;in sterile saline	LED; 455 nm; 22 mW/cm²	18 J/cm²;	complete (6 log_{10})
					10 µM/DS			25.5 J/cm²;	
					20 µM/DS			37.5 J/cm²	
			ATCC 33591 (MRSA)		20 µM/DS			37.5 J/cm²/	complete (6 log_{10})
196	CUR	*L. innocua* (1.3×10^7)	NCTC 11288	B	0.6 mg/L/DS	30 min; in ringer solution	LED; 400-500; 150 mW/cm²	270 J/cm²/30 min	4.4×10^3 CFU/mL
					3.7 mg/L/DS				1.7×10^2 CFU/mL
				P	0.6 mg/L/DS				5.3 log
					3.7 mg/L/DS				6.1 log
	CUR rich extract			B	0.6 mg/L;/DS;				1.4×10^5CFU/mL (2 log)
					1.1 mg/L;/DS				1.1×10^4 CFU/mL (3.1 log)
				P	0.6 mg/L/DS;				3.5 log
					1.1 mg/L/DS				5.4 log
155	CUR	*E. faecalis* (10^6)	A197A	P	2.5 µM (in a 5% DMSO preparation)/DS	30 min/PBS buffer	Light-polymerisation unit equipped with three fluorescent tubes emitting	0.5 J/cm²/−	99.8 %

		S. intermedius(10^6)	ATCC 27335				blue light; 430 nm; 17.3 mW/cm^2		
		E. coli (10^6)	ATCC 25922		25 μM (in a 5% DMSO preparation)/DS			30 J/cm^2/—	~ 100 %
197	CUR	*Lactobacilli* (10^8)	ATCC 4646	P	10 μM/L/DS	15 min/0.9% NaCl	Dental light source; 380-500 nm; —	—/240 sek	100%
154	CUR	*E. faecalis* (10^8)		P	20 μM/DS	5 min/ NaCl	LED; 450 nm; 100 mW/cm^2	5 min	41%
157	CUR	*S. mutans*(10^9)	OMZ 918	P	2 μM/DS	5 min/0.9% NaCl	Dental QTH lamp; 360-550 nm; 450 mW/cm^2	54 J/cm^2/120 s	95%
				B	60 μM/DS	10 min/0.9% NaCl			44 % (± 12 %)
159	CUR	*S.mutans* (10^7)	ATCC 25175	P	4000 μM	1 min/0.9% NaCl	LED/ 450 ±30 nm;	72 J/cm^2/300 s	5.3 $\log_{10}$
							240.1 mW/cm^2		
158	CUR	*S.mutans* (~2×10^9)	ATCC 700610 UA 159	P	0.75 mM/ 0.90 $\log_{10}$ reduction	1 min/PBS buffer	LED; 420 ±20 nm; 95.5 mW/cm^2	72 J/cm^2/4 min	complete (5.97 $\log_{10}$)

156	CUR	*E. faecalis* (5×10^{6})	135737	P	10 µM/DS	30 min/PBS buffer	dental quartz—tungsten—halogen; 450 nm	240 s	complete
				B					complete
95	Lyophylised extract of Solanumverbascifolium flower	*S. epidermidis* (3×10^{8})		P	0,05 mg/mL/DS	60 min/PBS buffer	ELH tungsten halogen GE Quartzline lamps with a reflector; 23 mW/cm^{2}	55 J/cm^{2}/40 min	4 log$_{10}$
						10 min/PBS buffer	Sunlight; 120 mW/cm^{2}	864 J/cm^{2}/120 min	complete (> 8 log$_{10}$)
165	Psoralen	*E. coli* (~ 10^{7})	CN-13 (ATCC 700609)	P	—	+ Lime Slurry	solar exposures	30-minute	> 6.1 logs
						+ Lime Juice			5.6 log
198	Protochlorophyllide (a biosynthetic intermediate during bacteriochlorophyll or chlorophyll biosynthesis), extract from Rhodobactercapsulatus ZY5;	*S. aureus* (~10^{7})	DSM 4910	P	10 mg/L	—/LB medium	60W tungsten lamp; 700 Lux; 0.1 mWcm^{2}	1.08 J/cm^{2}/3 h	7.1 log$_{10}$
		B. subtilis (~10^{8})	DSM 618					1.08 J/cm^{2}/3 h	5.1 log$_{10}$
		L. monocytogenes (~ 10^{8})	DSM 20600					1.08 J/cm^{2}/3 h	3.1 log$_{10}$
								1.08 J/cm^{2}/5.5 h	complete
		Yersinia pseudotuberculosis (~ 10^{7})	YPIII		10 mg/L Pchlide + 50 mg/L PMBN/ DS			0.72 J/cm^{2}/2 h	7.0 log$_{10}$
		E. coli (~ 10^{7})	DH10B		10 mg/L Pchlide + 20 mg/L PMBN/ DS			0.54 J/cm^{2}/1.5 h	8.2 log$_{10}$
176		*S. aureus* (5×10^{6})	ATCC8096		10^{-5} M/DS	1 h /NaCl;		—/1 h	3.1 log$_{10}$

	Sodium chlorophyllin (not copperized chlorophyll sodium salt); (Chl)	*B. cereus* (5×10^6)	ATCC9634			on plate count agar	Halogen photooptic lamp (1000 W);		3.1 log_{10}
		B. subtilis (5×10^6)	ATCC1904						4.2 log_{10}
93	Chl	*L. monocytogenes* (10^7)	ATC$_{L3}$C 7644	P	7.5×10^{-7} M/DS	2 min; in PBS buffer	LED; 400 nm; 20 mW/cm^2	6 J/cm^2/5 min	complete (7 log_{10})
		B. cereus (10^7)	ATCC 12826		7.5×10^{-7} M/DS			2.4 J/cm^2/2 min	
					7.5×10^{-8} M/DS			6 J/cm^2/5 min	
		S. enterica (10^7)	DS88(SL5676 Smr Plm32)		7.5×10^{-5} M/DS	60 min; in PBS buffer		24 J/cm^2/20 min	1.6 log_{10}
178	Chl	*L. monocytogenes* (10^7)	ATC$_{L3}$C 7644	P	7.5×10^{-7} M/DS	2 min/PBS buffer	LED; 405 nm; 20 mW/cm^2	6 J/cm^2/5 min	complete (7 log_{10})
		L. monocytogenes(10^7)	56 Ly					36 J/cm^2/30 min	
179	Chl	*L. monocytogenes* (~ 10^7)	ATC$_{L3}$C 7644	B	1.5×10^{-5} M;	22 min; on the surface of packaging samples	LED; 405 nm; 12 mW/cm^2	3.6 J/cm^2/5 min	1.5 log_{10}
					1.5×10^{-4} M				4.5 log_{10} (to undetectable level)

		B. cereus (~ 10^7)	SV 90	P	7.5×10^{-7} M/DS	2 min; PBS buffer		21.6 J/cm^2/30 min	~3.5 $\log_{10}$
173	Chl	*S. aureus* (10^8)	CECT 86	P	–; PS immobilized in gellatin films. It was added to the solution at 80 µg/mL./DS	0 min/on the surface of TSA	quartz/halogen lamps; white light; LED; 470 nm; –	30,000 lux/5 min	5 $\log_{10}$
		L. monocytogenes (10^8)	CECT 934						~5 $\log_{10}$
		E. coli (10^8)	CECT 515						Not susceptible
		Salmonella spp. (10^8)	CECT 409						Not susceptible
	Chlorophyllin sodium copper salt (CuChlNa)	*S. aureus* (10^8)	CECT 86						4 $\log_{10}$
		L. monocytogenes (10^8)	CECT 934						~ 4 $\log_{10}$
		E. coli (10^8)	CECT 515						Not susceptible
		Salmonella spp. (10^8)	CECT 409						Not susceptible
199	CuChlNa	*S. aureus* (~ 5×10^8)	ATCC 25923	P	5 µM/DS	30 min/NaCl	LED; 405 nm; 8.3 mW/cm^2	30 J/cm^2/60 min	Complete
		E. coli (~ 5×10^8)	ATCC 25922	P	5 µM/DS+ DMSO	120 min/NaCl			Not susceptible
180	Chl	*L. monocytogenes* (10^5)	CECT 911 (ATCC 19112)	P	1% (incorporated with PE)/DS	0 min/on the surface of TSA	500 W Haloline 64702 halogen lamps (Osram); infrared region;	15 min	0.65 $\log_{10}$

							I		
					1% (incorporated with PVOH)/DS				7,25 $\log_{10}$
					1% (incorporated with HPMC)/DS				7.01 $\log_{10}$
					1% (incorporated with G)/DS				7.09 $\log_{10}$
		E. coli (10^5)	CECT 434 (ATCC 25922)		1% (incorporated with PE)/DS	on the surface of TSA plates; 40 mM EDTA	50 W 81.575/Dia LED lights (Electro DH); 450 nm and 550 nm; I		0.67 $\log_{10}$
					1% (incorporated with PVOH)/DS				7.47 $\log_{10}$
					1% (incorporated with HPMC)/DS				7.04 $\log_{10}$

					1% (incorporated with G)/DS				7.04 log_{10}
182	Chl(E)	*E. coli*; OD_{590} = 0.1 (corresponding to 0.8×10[8])	DH5*α*	P	10 mg/L/ DS	LB medium	LED; 12 mW/cm[2]	129.6 J/cm[2]/180 min	Not susceptible
					10 mg/L Chl+0.1 μg/L colistin; ~ OD 0.2				~ OD 0.5
		S. TyphymuriumOD_{590} = 0.1 (corresponding to 0.8×10[8])	Ames9274		0.5 mg/L/ DS				Completely inhibited the growth
					0.5 mg/L Chl+0.1 μg/L colistin;/ ~ OD 0.2				Completely inhibited the growth
94	Chl(preparated from frozen spinach)	*E. coli* (10[8])	DH5*α*	P	25 mg/L/DS	0 min/LB medium	LED; 12 mW/cm[2]	30-180 min	Not susceptible
		E. coli (10[6])	NR698 (MC4100 *lptD4213*)		5 mg/L/DS			60 min	Completely inactivated
		B. subtilis (10[8])	168 (*trpC2*)		0.5 mg/L/DS			60 min	Completely inactivated

200	Chl	*E. coli* (10^7)	O157:H7	P	1×10^{-5} M/DS	60 min;	LED; 405 nm; 9.6 mW/cm^2 from top and 11.5 mW/cm^2 from bottom	38 J/cm^2/30 min	~3 log_{10}
	Chl + ZnO nanoparticles.				1×10^{-4} M/DS	15 min with ZnO + 15 min with Chl/PBS buffer			4.5 log_{10}
177	Chl	*L. monocytogenes* (10^7)	ATC$_{L3}$C 7644	P	7.5×10^{-7} M/DS	2 min/PBS buffer	LED; 400 nm; 20 mW/cm^2	3.6 J/cm^2/5 min	7 log_{10}
		S. enterica (10^7)	DS88(SL5676 Smr Plm32)		7.5×10-5 M/DS			28.8 J/cm^2/40 min	~2.2 log_{10}
	Chl + 5-aminolevulinic acid hydrochloride (Ala)	*L. monocytogenes* (10^7)	ATC$_{L3}$C 7644		7.5×10^{-7} M Chl + 7.5×10^{-3} M Ala/DS	2 min; in PBS buffer		17.3 J/cm^2/30 min	undetectable level (~ 7.5 log_{10})
		S. enterica (10^7)	DS88(SL5676 Smr Plm32)		7.5×10^{-5} M Chl + 7.5×10^{-3} M Ala/DS	60 min/PBS buffer			undetectable level (~ 7.5 log_{10})
201	Chl-KCHS (chitosan)	*S. enterica* (10^8)	DS88(SL5676 Smr Plm32)	P	0.001% - 0.1%/DS	0-4 h/ LB medium	LED; 405 nm; 9.6 mW/cm^2	17.3 J/cm^2/30 min	Not susceptible
		L. monocytogenes (~ 10^8)	ATC$_{L3}$C 7644		0.001%-0.1%/ 5.4 log_{10} -after 4 h incubation	0-4 h/LB medium		2.9 J/cm^2/5 min	complete (8 log_{10})
202/175	Chl-^{12}CHSconjugate	*S. enterica* (10^7)		P	1.5×10^{-5} M - 0.1%/2.15 log_{10} -	0-120 min/0.9% NaCl	LED; 405 nm;	38 J/cm^2/30 min	complete (7 log_{10})

			DS88(SL56 76 Smr Plm32)		after 2 h incubation		21 mW/cm^2		
181	Chl-CHS	*L. monocytogenes* (~ 10^7)	ATC$_{L3}$C 7644	P	0.0001%-0.001%/ 2.1 log$_{10}$ - after 2 min incubation	2 min/0.9% NaCl	LED; 405 nm; 9.6-11.52 mW/cm^2	2.9 J/cm^2/2 min	7.01 log$_{10}$
		E. coli (~ 10^7)	157:H7		0.001% - 0.1%/ 0.5 log$_{10}$ - after 1 h incubation	60 min/0.9% NaCl		38 J/cm^2/30 min	4.5 log$_{10}$
								76 J/cm^2/60 min	7 log$_{10}$
96	water extract of the calyces of *Hibiscus sabdariffa* L (cHs WE)	*E. coli* (~ 10^7)	Nissle 1917	P	1 mg/mL	in d H_2O	Slide Projector—Zett Royal II afs; 420 nm; 10 mW/cm^2	12 J/cm^2/20 min	complete -8.5 log
		B. subtilis (10^8)	DB 104		0.0625 mg/mL				2.2 log
	methanol extract of the calyces of *H. Sabdariffa* (cHs ME)	*E. coli* (~10^7)	Nissle 1917		1 mg/mL				4.8 log
		B. subtilis (10^8)	DB 104		0.0625 mg/mL				complete- 8.5 log

[1]P - planktonic cells; [2]B - biofilms; [3]DS- doesn't show;[4]PBS – phosphate-buffered saline; [5]LED- light emitting diode;[6]—no information; [7]NS – not susceptible; [8]LB - Luria Bertani;[9]TSB - tryptic soy broth;[10]NB - nutrient broth;[11]FUM - fluid universal medium; [12]CHS- chitosan
